# Three new species of *Fufius* Simon, 1888 (Araneae, Cyrtaucheniidae) from Brazil with the redescription of *Fufius funebris* Vellard, 1924 and description of the female of *Fufius lucasae* Guadanucci & Indicatti, 2004

**DOI:** 10.3897/zookeys.352.6189

**Published:** 2013-11-19

**Authors:** Diego Ribeiro Migueis Ortega, Roberto Hiroaki Nagahama, Paulo Cesar Motta, Rogério Bertani

**Affiliations:** 1 Instituto Butantan, Laboratório Especial de Ecologia e Evolução, Av. Vital Brazil, 1500, 05503-900, São Paulo – SP, Brazil; 2 Universidade de Brasília, Departamento de Zoologia, 70910-900, Brasília – DF, Brazil

**Keywords:** Brazilian Atlantic rainforest, cerrado, mygalomorph, new species, taxonomy

## Abstract

The mygalomorph neotropical genus *Fufius* Simon, 1888 comprises ten species, distributed from Guatemala in Central America to southeastern Brazil, in South America. Most of the species were described from northern South America, in the Amazonian region. Only *F. funebris* Vellard, 1924 and *F. lucasae* Guadanucci & Indicatti, 2004 are known from regions more to the south of the continent. Herein we describe three new Brazilian species, *Fufius minusculus* **sp. n.** and *F. jalapensis* **sp. n.** from the state of Tocantins, and *F. candango* **sp. n.** from Distrito Federal. The female of *F. lucasae* is described for first time and the male and female of *F. funebris* are redescribed based on specimens collected at the type locality.

## Introduction

The genus *Fufius* comprises ten species distributed from Guatemala (*Fufius atramentarius* Simon, 1888 – type species) southwards to South America: Colombia – *Fufius annulipes* (Mello-Leitão, 1941), Ecuador – *Fufius ecuadorensis* (Simon, 1892), Bolivia – *Fufius lanicius* (Simon, 1892), Trinidad – *Fufius antillensis* (F.O.P-Cambridge, 1898), and Brazil – *Fufius albovittatus* (Simon, 1891), *Fufius auricomus* (Simon, 1891), *Fufius funebris* Vellard, 1924, *Fufius lucasae* Guadanucci & Indicatti, 2004, and *Fufius striatipes* (Drolshagen & Bäckstam, 2009). Except for *Fufius funebris* and *Fufius lucasae*, all other Brazilian species were described from specimens collected in the Amazon. *Fufius funebris* was described by Vellard (1924) from Catalão, state of Goiás, Brazil. These types are supposed to be lost (Guadanucci and Indicatti 2004), which led these authors to redescribe the species with a female collected at the type locality and a male from the Distrito Federal (ca. 260 km northwards). In the same paper, Guadanucci and Indicatti (2004) describe *Fufius lucasae*, based only in males, from the state of São Paulo.

*Fufius* has a very controversial taxonomic position. This originally monotypic genus was formerly included by Simon (1888, 1891) in his Ctenizinae and, subsequently, transferred to his Diplurinae (Simon 1892a, b). Other species, e. g., *Fufius ecuadorensis*, was described in the genus *Phrissaecia* Simon, 1892 (Aporoptycheae, Ctenizinae), and posteriorly this genus was synonymized with *Fufius* by the same author (Simon 1903). In his revision and cladistics of mygalomorph genera, Raven (1985) considered *Fufius* unequivocally to belong in the Cyrtaucheniidae, where it is presently included. However, posterior morphological cladistic analyses performed by Goloboff (1993; 1995) suggest that the Cyrtaucheniidae is paraphyletic. More recent analysis carried out using morphological and molecular data (Bond et al. 2012) suggests a polyphyly of Cyrtaucheniidae. The same analysis show that *Fufius* is undoubtedly more related to the Nemesiidae. Besides the difficulty in stablishing the taxonomic position of *Fufius*, most species in the genus were described either with a single female or male specimen, limiting the information on the intra and interespecific morphological variability of the genus.

Herein we collaborate to a better knowledge of this little known genus, describing three new *Fufius* species from non-amazonian Brazil, two from the state of Tocantins, and one from the Distrito Federal. The female of *Fufius lucasae* is described for first time and the male and female of *Fufius funebris* are redescribed based on specimens collected at the type locality. The male of *Fufius funebris* redescribed by Guadanucci and Indicatti (2004) was misidentified by those authors, and corresponds to the new species herein described from Distrito Federal.

## Methods

All measurements are in millimeters. Total length does not include chelicerae or spinnerets. Leg and palp measurements were taken from the dorsal aspect of the left side (unless appendages were lost or obviously regenerated) with a Mitutoyo digital caliper, which had an error of 0.005 mm, rounded up to two significant decimals. A Leica LAS Montage and LAS 3D module mounted on a Leica M205C dissecting microscope were used for image capture and measurements of other spider structures. Spermathecae were cleared by means of immersion in clove oil. Abbreviations: ALE = anterior lateral eyes, AME = anterior median eyes, ap = apical, d = dorsal, ITC = inferior tarsal claw, p = prolateral, PLE = posterior lateral eyes, PLS = posterior lateral spinnerets, PME = posterior median eyes, PMS = posterior median spinnerets, r = retrolateral, SB = spermatheca bulb, spnf = spiniform, STC = superior tarsal claws, SS = spermatheca stalk, and v = ventral. Terminology for spermatheca follows Coyle (1995), for spination follows Petrunkevitch (1925).

Specimens are deposited in the following institutions: DZUB, Departamento de Zoologia, Universidade de Brasília, Brasília (Paulo C. Motta); IBSP, Instituto Butantan, São Paulo (Yara Cury); INPA, Instituto Nacional de Pesquisas da Amazônia, Manaus (Ana L. Tourinho); MNHN, Muséum national d’Histoire naturelle, Paris (Christine Rollard); MZSP, Museu de Zoologia, Universidade de São Paulo, São Paulo (Ricardo Pinto-da-Rocha).

Geographical coordinates: primary sources are between round brackets and secondary sources (Google Earth©) are between square brackets. The coordinates from the secondary source were obtained from the center of the municipality cited in the specimens labels and are in DMS (Degrees, Minutes and Seconds) format rounded off to minutes.

Additional type material examined: *Fufius albovittatus* (Simon, 1891), holotype male, MNHN 9666, from Brazil, Manaus, Haunwell leg.; *Fufius atramentarius* Simon, 1888, holotype female, MNHN 4945, from Guatemala, Perrot leg.; *Fufius ecuadorensis* (Simon, 1892), holotype female, Ecuador, Loja; *Fufius striatipes* (Drolshagen & Bäckstam, 2009) holotype male, Brazil, state of Amazonas, Manaus, Tarumã Mirim, 03°06'00"S, 60°01'48"W, J. Adis leg., February 1982 (INPA 3507), examined by photographs.

## Taxonomy

### Cyrtaucheniidae Simon, 1889
*Fufius* Simon, 1888

*Fufius* Simon, 1888: 213 (type species by monotypy *Fufius atramentarius* Simon, 1888).

*Hapalothele* (in part: *Hapalothele albovittata* Simon, 1891: 306; *Hapalothele auricomus* Simon, 1891: 305; *Hapalothele lanicia* Simon, 1892: 283).

*Brachythele* (in part: *Brachythele antillensis* F.O.P.-Cambridge, 1898: 899).

*Phrissaecia* Simon, 1892: 274 (type species by monotypy *Phrissaecia ecuadoriensis* Simon, 1892). First synonymized by Simon 1903: 967.

*Hermorhachias* Mello-Leitão, 1941: 234 (type species by original designation *Hermorhachias annulipes* Mello-Leitão, 1941). First synonymized by Raven 1985: 134.

*Metriura* Drolshagen & Bäckstam, 2009: 365 (type species by monotypy *Metriura striatipes* Drolshagen & Bäckstam, 2009). First synonymized by Bertani et al. 2012.

#### Fufius funebris

Vellard, 1924

http://species-id.net/wiki/Fufius_funebris

Figs 1 –11 , 45

Fufius funebris Vellard, 1924: 153; Guadanucci and Indicatti 2004: 256, fig. 7 (redescribed male and female, male misidentified).

##### Diagnosis.

**Figures 1–6.**
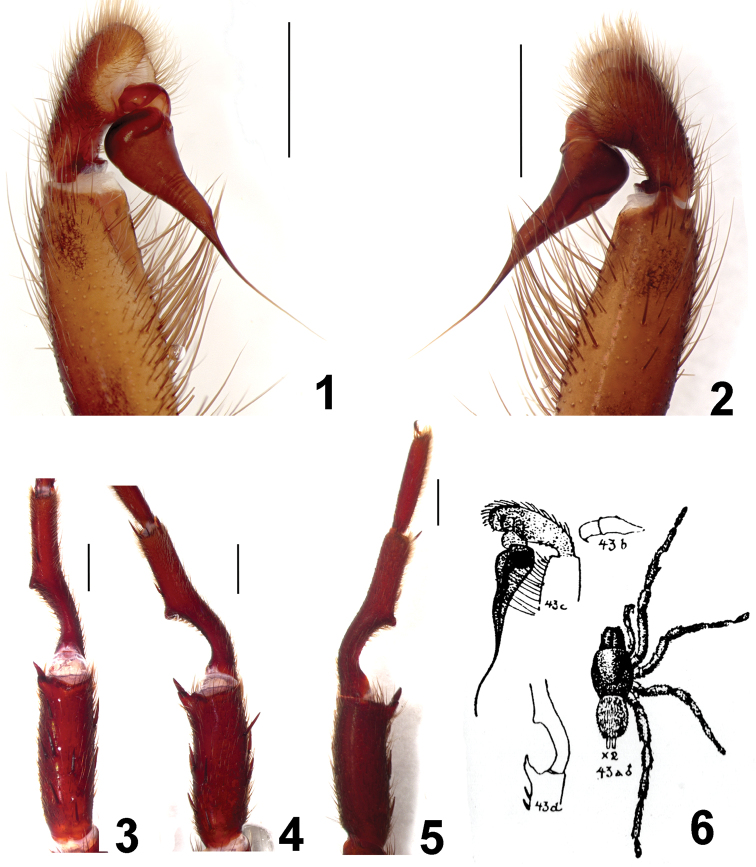
*Fufius funebris*. Male (DZUB 2531-1) **1–2** right palpal bulb **1** retrolateral view **2** prolateral view **3–5** right leg I tibial spur **3** ventral view **4** prolateral view **5** retrolateral view **6** reproduction of Vellard’s 1924 *Fufius funebris* plate. Scale bar = 1mm.

The male differs from those of all other species in the genus by the characteristic very long embolus having a subtle constriction on its middle (Figs 1–2). The female differs from those of all other species by the spermathecae having spiraled stalks (Fig. 9).

##### Types.

Syntypes, 3 males and 8 females, should be deposited in Instituto Vital Brazil, Niterói; 1 female, 1 male in the personal collection of Jean Vellard. Types lost (Guadanucci and Indicatti 2004).

##### Additional material examined.

BRAZIL: *Goiás*: Catalão [18°09'S, 47°56'W], 2 males, 1 female, 20 October 2001, P.C. Motta, with pitfall trap (DZUB 2531); 1 female, 22 October 2001, P.C. Motta, on termite mound (DZUB 435); *Minas Gerais*: Uberlândia [18°54'S, 48°15'W], Fazenda do Glória, 1 female, P. C. Motta (IBSP 11102); 3 females, 1 immature, 14 November 1990, P.C. Motta (DZUB 4470); Fazenda São José, 1 female, 15 July 1992, P.C. Motta (DZUB 318).

##### Male redescription

(DZUB 2531–1). Total length: 9.70. Carapace 5.89 long, 4.63 wide, chelicerae 2.87 long, 1.48 wide. Palp: femur 2.92, patella 1.65, tibia 2.25, tarsus 0.95, total 7.77. Legs (femur, patella, tibia, metatarsus, tarsus, total): I: 4.18, 2.65, 3.10, 3.55, 2.28, 15.76. II: 4.07, 2.31, 2.70, 2.97, 2.08, 14.13. III: 3.31, 2.01, 2.17, 2.96, 1.77, 12.22. IV: 3.87, 2.27, 3.25, 4.09, 1.79, 15.27. Mid-widths (lateral): femora I–IV = 1.01, 0.92, 1.10, 1.13, palp = 0.90; patellae I–IV = 1.11, 0.94, 1.02, 1.07, palp = 0.83; tibiae I–IV = 1.31, 0.86, 0.68, 0.84, palp = 0.83; metatarsi I–IV = 0.78, 0.54, 0.63, 0.52; tarsi = 0.52, 0.38, 0.45, 0.48, palp = 0.75. Abdomen 5.90 long, 4.10 wide. Spinnerets: PMS, 0.8 long, 0.31 wide, 0.46 apart; PLS, 1.25 basal, 0.96 middle, 0.96 distal; mid-widths (lateral), 0.61, 0.57, 0.4 respectively. Carapace (Fig. 10): length to width 1.27. Fovea strongly recurved, 0.93 wide. Eyes: tubercle 0.38 high, length 0.71, width 1.17. Clypeus 0.14. Anterior and posterior eye row recurved. Eyes sizes and inter-distances: AME 0.30, ALE 0.30, PME 0.15, PLE 0.22, AME–AME 0.21, AME–ALE 0.09, PME–PME 0.61, PME–PLE 0.02, ALE–PLE 0.10, AME–PME 0.14, ALE–ALE 0.77, PLE–PLE 0.82. Eye group width 1.16, length 0.59. Maxillae (Fig. 11) 2.04 long, 1.34 wide. Cuspules: 61 spread over ventral inner heel. Labium: 0.95 long, 1.02 wide, with 4 cuspules. Labio-sternal groove shallow, flat with two large sigillae. Sternum: 3.16 long, 2.56 wide. Three pairs of sigillae, first, second rounded, posterior ovals, all one diameter from margin. Chelicerae: basal segment with 9 teeth. Legs: leg formula: I IV II III. Scopula: tarsi I–IV scopulate. Metatarsi I–II 1/3 scopulate. Spines: palp: femur p0-0-1ap, patella p1-0-1; leg I: femur d1-1-2(spnf), patella v1, p2, tibia v2-2-1, p1-5-0, metatarsus v0-1-1ap; leg II: femur d1-2-1(spnf), patella v1, p2, tibia v4-3-2(2ap), metatarsus v5-3-2ap, p0-1-0; leg III: femur d1-2-1(spnf), patella p3, r1, tibia d1-0-0, v2-4-2ap, p0-0-1, r0-0-1, metatarsus d3-2-2, v3-2-2ap, p0-2-0, r0-0-1ap; leg IV: femur d2-1-1(spnf), tibia v2-2-2ap, r1-0-1, metatarsus d0-1-2ap, v2-3-3(2ap), r0-0-1ap, p1-1-1ap. Preening-comb: absent on retrolateral tip of metatarsus IV. ITC smooth, STC with two rows of 5–8 teeth on both margins on all legs. Palp: embolus 1.76 in length. Embolus (Figs 1–2) basal, middle and distal width of 0.34, 0.03, 0.01, respectively. Tegulum 0.49 long. Tibial spur (Figs 3–5) formed by single branch 0.63 long, 0.42 wide, on retrolateral margin, with apical spine. Color pattern: carapace black with some long golden setae. Sternum brown, labium, maxillae dark brown. Abdomen dorsally black with rounded whitish spot on anterior central region, brown punctuations on remaining areas, ventrally with two large whitish areas on lateral regions. Spinnerets light brown with brown setae. Leg I black with some golden setae. Legs II–IV dark brown with brown spots, femur darker, with several golden setae, coxae brown with some brown setae.

**Figures 7–11.**
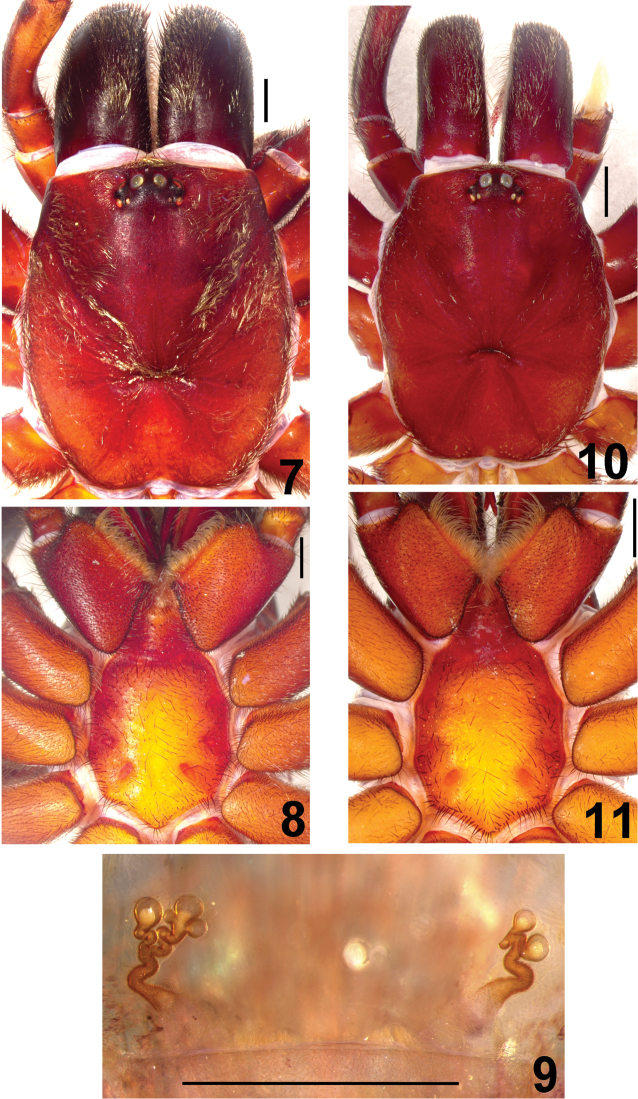
*Fufius funebris* **7–9** female (DZUB 2531-2) **7** carapace **8** sternum, labium and maxillae **9** spermathecae, dorsal view **10–11** male (DZUB 2531-1) **10** carapace **11** sternum, labium, and maxillae. Scale bar = 1mm.

##### Female redescription

(DZUB 2531-2). Total length: 17.46. Carapace 7.24 long, 6.13 wide, chelicerae 3.31 long, 2.34 wide. Palp: femur 3.54, patella 1.94, tibia 2.00, tarsus 2.32, total 9.80. Legs (femur, patella, tibia, metatarsus, tarsus, total): I: 4.65, 3.34, 3.31, 3.56, 2.36, 17.22. II: 4.40, 3.14, 2.95, 3.21, 2.17, 15.87. III: 4.06, 2.66, 1.86, 2.95, 2.06, 13.59. IV: 5.35, 2.93, 3.66, 4.12, 2.14, 18.20. Mid-widths (lateral): femora I–IV = 1.28, 1.15, 1.43, 1.33, palp = 0.80; patellae I–IV = 1.40, 1.29, 1.31, 1.31, palp = 1.14; tibiae I–IV = 1.28, 0.98, 1.13, 1.05, palp = 1.16; metatarsi I–IV = 0.72, 0.72, 0.52, 0.66; tarsi = 0.49, 0.49, 0.47, 0.57, palp = 0.84. Abdomen (damaged) ca. 9.84 long, 6.74 wide. Spinnerets: PMS, 1.03 long, 0.48 wide; PLS, 1.82 basal, 1.07 middle, 1.71 distal; mid-widths (lateral), 0.96, 0.87, 0.72 respectively. Carapace (Fig. 7): length to width 1.18. Fovea recurved, 1.77 wide. Eyes: tubercle 0.44 high, length 1.16, width 1.59. Clypeus 0.03. Anterior and posterior eye row recurved. Eyes sizes and inter-distances: AME 0.42, ALE 0.34, PME 0.17, PLE 0.26, AME–AME 0.18, AME–ALE 0.14, PME–PME 0.85, PME–PLE 0.06, ALE–PLE 0.21, AME–PME 0.25, ALE–ALE 1.02, PLE–PLE 1.22. Eye group width 1.58, length 0.74. Maxillae (Fig. 8) 2.71 long 2.77 wide. Cuspules: ca. 57 spread over ventral inner heel. Labium: 1.28 long, 1.46 wide, with 3 cuspules. Labio-sternal groove shallow, flat with two large sigillae. Sternum: 4.29 long, 3.35 wide. Three pairs of sigillae, first rounded, second, third ovals, all one diameter from margin. Chelicerae: basal segment with 9 teeth. Legs: leg formula: IV I II III. Scopula: tarsi I–II scopulate. Metatarsi I 2/3, II 1/2 scopulate. Spines: palp: femur, patella 0, tibia v2-2(spnf)-2ap, p1-0-1ap(spnf), tarsus v2-0-0; leg I: femur, patella 0, tibia v0-0-2ap(spnf), metatarsus v0-1-2ap; leg II: femur, patella 0, tibia v0-1-2ap(spnf), metatarsus v2-2-2ap, p0-0-1ap; leg III: femur 0, patella p4, r1, tibia v2-2-2(spnf), p0-0-1ap, metatarsus d1-1-1ap, p0-2-1, v2-2-2ap; leg IV: femur, patella 0, tibia v3-2-1ap(spnf), r0-0-1(spnf), metatarsus d0-0-1ap, r0-1-1ap, v2-1-2(1ap), r0-1-1. Preening-comb: absent on retrolateral tip of metatarsus IV. Palp with a single claw having 6 small teeth on internal margin. ITC smooth, STC with two rows of 5–8 teeth on both margins on all legs. Spermathecae: two spermathecae having a narrow, spiraled stalk, giving origin to two spiraled branches with 1–3 bulbs. Color pattern: as in male, except legs black with two (femur, patella, tibia) or one (metatarsus, tarsus) brown stripes (lacking setae) on dorsal area. Abdomen ventrally without two large whitish areas on lateral regions.

##### Remarks.

Vellard (1924) described *Fufius funebris* based on 3 males and 8 females from Catalão, Goiás, Brazil. The types should be deposited in the collection of Instituto Vital Brazil, Niterói, but were not found and considered lost (Guadanucci and Indicatti 2004). These authors redescribed this species based on a female from the type locality and a male from Brasilia, Distrito Federal, ca. 260 km northwards. However, additional specimens we obtained from type locality, both male and female, and show that the female was correctly identified by Guadanucci and Indicatti (2004), but not the male. Vellard (1924) published a figure showing in detail the *Fufius funebris* embolus morphology (Fig. 6), which is very long and has a median constriction. An embolus tapering to its tip can be easily distinguished from the Guadanucci and Indicatti (2004) drawings, as shown in their figs 1–3. Conversely,the bulbs of the specimen we obtained from type locality (Figs 1–2) fits very well with the figure of Vellard (1924) (Fig. 6) and can be assigned without doubts to *Fufius funebris*. The male of the species Guadanucci and Indicatti (2004) attributed to *Fufius funebris* belongs to a new species, described below as *Fufius candango* sp. n.

##### Distribution.

Brazil: states of Goiás (Catalão) and Minas Gerais (Uberlândia) (Fig. 45).

#### Fufius lucasae

Guadanucci & Indicatti, 2004

http://species-id.net/wiki/Fufius_lucasae

**Figures 12–15.**
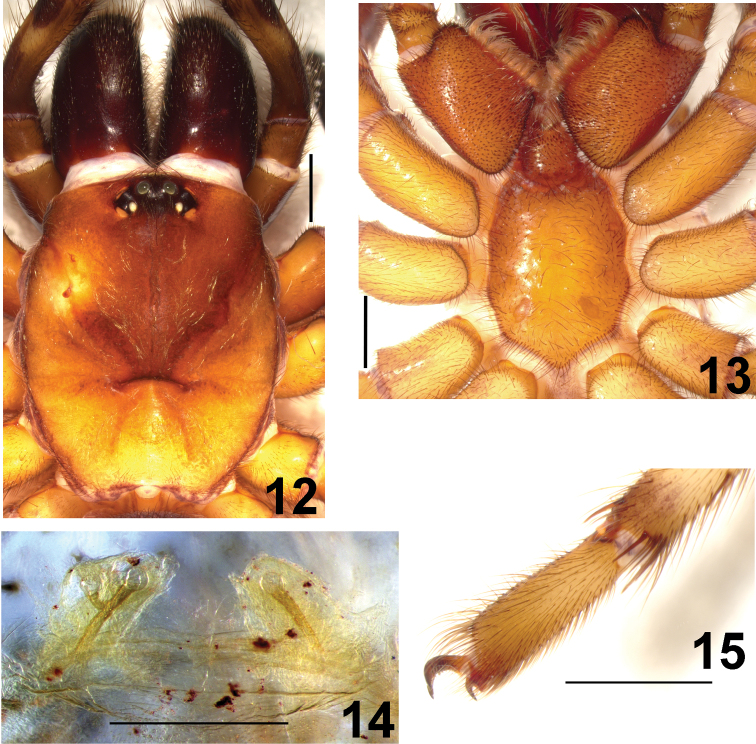
*Fufius lucasae*, female (DZUB 8021) **12** carapace **13** sternum, labium and maxillae **14** spermathecae, dorsal view **15** left metatarsus IV, retrolateral, showing preening-comb. Scale bar = 1mm.

Figs 12–15 , 44 , 45

Fufius lucasae Guadanucci & Indicatti, 2004: 257, figs 8–13.
Fufius lucasi : Platnick 2013. (N.B. matronym for Sylvia Lucas).

##### Diagnosis.

The male differs from those of *Fufius funebris* by the shorter and tapering embolus (see figs 8–10 in Guadanucci and Indicatti 2004), from *Fufius jalapensis* sp. n. by having spines on tibia I, from *Fufius minusculus* sp. n. by having a carapace less than 1.5 times longer than the width, from *Fufius albovittatus* by metatarsus I being very curved at the basis and having only 2 spines on tibia I, from *Fufius candango* sp. n. by the preening comb on metatarsus IV formed by macrosetae instead of small spines, and from *Fufius striatipes* by having only 2 spines on tibia I instead of 11. The female differs by the spermatheca stalk being straight and narrow, and giving origin to 1–2 lobes (Fig. 14).

##### Types.

Holotype male from Brazil, *São Paulo*, São Paulo, Parque Estadual da Serra da Cantareira, Núcleo da Pedra Grande [23°26'S, 46°38'W], December 2000, C. C. Aires et al., with pitfall trap (MZSP 23231), examined; paratypes: 2 males, same collector and date (MZSP 23226, IBSP 10993), examined; male from Brazil, *São Paulo*, São Paulo, Parque Estadual do Jaraguá [23°27'S, 46°46'W], 17 January 2004, R. P. Indicatti (IBSP 10952), not examined; 2 males from Brazil, *São Paulo*, Cotia, Caucaia do Alto, Reserva Florestal do Morro Grande [23°39'S, 46°57'W], December 2002, J. M. B. Ghelleri (MZSP 22017), examined.

##### Additional material examined.

BRAZIL: *São Paulo*: São Paulo, Parque Estadual da Serra da Cantareira, Núcleo da Pedra Grande [23°26'S, 46°38'W], 1 male, December 2000, C. C. Aires et al., with pitfall trap (MZSP 23225); 1 male, same collector and date (MZSP 23227); 1 male, same collector and date (MZSP 23229); 1 male, same collector and date (MZSP 23230); 2 males, same collector and date (MZSP 23232); 1 male, same collector and date (MZSP 23233); 1 male, same collector and date (MZSP 23234); 1 male, same collector and date (MZSP 23235); 1 male, same collector and date (MZSP 23236); 1 male, same collector and date (MZSP 23237); Itapecerica da Serra [23°46'S, 46°50'W], 1 male, 16 May 2004, D.R.M. Ortega (DZUB 8018); 1 female, 1 immature, 2004, same collector, on a web on the trunk of a “Quaresmeira” tree (*Tibouchina* sp., Melastomatacea) (DZUB 8019); 1 female, 2005, same collector and date (DZUB 8020); 1 female, June 2006, same collector (DZUB 8021).

##### Female description

(DZUB 8021): Total length: 15.61. Carapace 6.49 long, 5.26 wide, chelicerae 3.40 long, 2.11 wide. Palp: femur 3.45, patella 1.84, tibia 2.00, tarsus 2.25, total 9.54. Legs (femur, patella, tibia, metatarsus, tarsus, total): I: 4.61, 2.70, 3.49, 3.69, 2.23, 16.72. II: 3.96, 2.66, 2.95, 2.83, 2.17, 14.57. III: 3.02, 2.33, 1.93, 2.48, 1.90, 11.66. IV: 4.25, 2.51, 3.69, 3.20, 1.91, 15.56. Mid-widths (lateral): femora I–IV = 1.04, 1.06, 1.29, 1.29, palp = 0.93; patellae I–IV = 1.05, 1.07, 1.16, 1.10, palp = 1.06; tibiae I–IV = 0.96, 1.04, 0.98, 1.02, palp = 0.97; metatarsi I–IV = 0.69, 0.62, 0.68, 0.63; tarsi = 0.62, 0.51, 0.47, 0.53, palp = 0.89. Abdomen 9.24 long, 5.87 wide. Spinnerets: PMS, 0.96 long, 0.48 wide, 0.63 apart; PLS, 1.43 basal, 0.94 middle, 1.08 distal; mid-widths (lateral), 0.86, 0.73, 0.48 respectively. Carapace (Fig. 12): length to width 1.23. Fovea recurved, 1.57 wide. Eyes: tubercle 0.58 high, length 0.88, width 1.75. Clypeus 0.05. Anterior and posterior eye row recurved. Eyes sizes and inter-distances: AME 0.27, ALE 0.34, PME 0.18, PLE 0.30, AME–AME 0.28, AME–ALE 0.251, PME–PME 0.78, PME–PLE 0.10, ALE–PLE 0.14, AME–PME 0.20, ALE–ALE 1.22, PLE–PLE 1.12. Eye group width 1.7, length 0.72. Maxillae (Fig. 13) 1.71 long, 2.42 wide. Cuspules: ca. 62 spread over ventral inner heel. Labium: 1.15 long, 1.21 wide, with 18 cuspules. Labio-sternal groove shallow, flat with two large sigillae. Sternum: 3.56 long, 2.67 wide. Three pairs of sigillae, first rounded, second, third ovals, all one diameter from margin. Chelicerae: basal segment with 8 teeth. Legs: leg formula: I IV II III. Scopula: tarsi I–II scopulate. Metatarsi I 2/3, II 1/3 scopulate. Spines: palp: femur, patella 0, tibia v2-4-3ap(spnf), tarsus v2-0-0; leg I: femur 0, patella p1, tibia v1-1-2ap(spnf), metatarsus v2-1-2ap; leg II: femur, patella 0, tibia v1-1-1ap(spnf), metatarsus v1-2-2ap, r0-0-1ap; leg III: femur 0, patella p2, tibia p1-1-0, v2-2-1ap(spnf), metatarsus d3-2-0, p0-2-1ap, v2-2-2ap, r0-1-0; leg IV: femur, patella 0, tibia v0-1-1(spnf), metatarsus d0-1-0, v2-3-2ap, p0-0-1ap. Preening-comb (Fig. 15): formed by 4 spiniform setae between two spines on retrolateral tip of metatarsus IV. Palp with single claw having 6 small teeth on internal margin. ITC smooth, STC with two rows of 4–6 teeth on both margins on all legs. Spermathecae (Fig. 14): two spermathecae having narrow, straight stalk, giving origin to one or two straigth branches ending in 1–2 bulbs. Color pattern (Fig. 42): carapace light brown with some long golden setae, sternum and coxae light brown, labium and maxillae dark brown. Abdomen dorsally black with rounded whitish spot on anterior central region, brown punctuations on remaining areas. Spinnerets light brown with dark brown setae. Legs yellow with brown spots on basal and apical region of femur, patella, tibiae, and metatarsus, in addition, a central spot on metatarsus.

##### Distribution.

Brazil: state of São Paulo (São Paulo, Cotia and Itapecerica da Serra) in the Brazilian Atlantic Forest (Fig. 45).

#### Fufius candango sp. n.

http://zoobank.org/EAB79FAB-20C4-4F6F-A4A4-73AF5FE4E052

http://species-id.net/wiki/Fufius_candango

Figs 16 –26 , 45

Fufius funebris : Guadanucci and Indicatti 2004: 256, figs 1–6 (male, misidentified).

##### Diagnosis.

Male and female differ from those of all other species by presence of preening comb on retrolateral tip of metatarsus IV formed by small spines (Fig. 24).

##### Etymology.

The specific name, candango, refers to the workers who were largely responsible for building Brazil’s capital, Brasilia, where the type specimens of the new species were collected.

##### Types.

Holotype male from Brazil, *Distrito Federal*, Brasília, campus da Universidade de Brasília [15°45'S, 47°52'W], 26 November 1996, E. Mamede, with pitfall trap (DZUB 709). Paratypes: male from Brazil, *Distrito Federal*, Brasília, Reserva do IBGE [15°56'S, 47°53'W], 06 December 1996, E. Mamede, with pitfall trap (DZUB 714); 2 females, junction of railway DF 140 with BR 251 [15°56S, 47°49'W], inside a burrow covered with silk strands in an embankment, 7 October 2006, P.C. Motta (DZUB 4492).

##### Additional material examined.

BRAZIL: *Distrito Federal*: Brasília, Reserva da Marinha [16°00'S, 47°57'W], 3 males, 29–31 October 1999, G.G. Montingelli, with pitfall trap (IBSP 8015).

##### Male description

**Figures 16–20.**
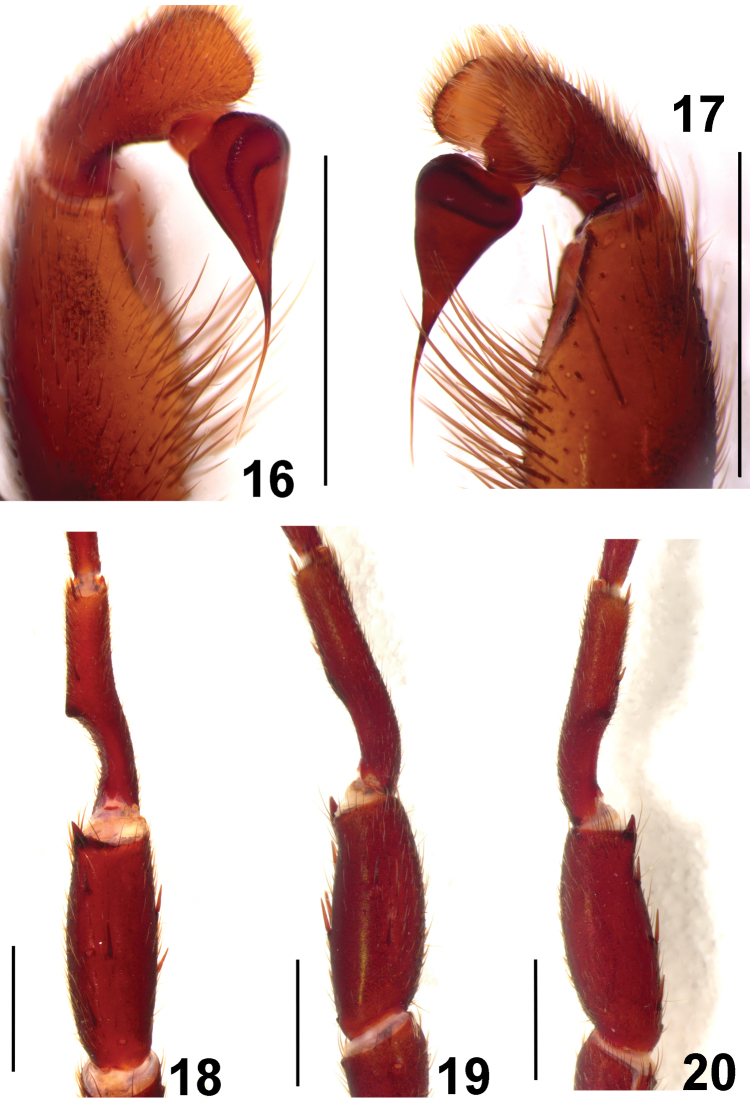
*Fufius candango* sp. n., holotype male **16–17** right palpal bulb **16** prolateral view **17** retrolateral view **18–20** right leg I tibial spur **18** ventral view **19** prolateral view **20** retrolateral view. Scale bar = 1mm.

(DZUB 709). Total length: 9.74. Carapace 4.77 long, 3.85 wide, chelicerae 3.70 long, 1.16 wide. Palp: femur 2.41, patella 1.24, tibia 1.49, tarsus 1.01, total 5.88. Legs (femur, patella, tibia, metatarsus, tarsus, total): I: 3.31, 2.01, 2.21, 2.84, 2.68, 13.05. II: 2.99, 1.68, 2.02, 2.35, 1.57, 10.61. III: 2.70, 1.40, 1.83, 2.27, 1.48, 9.68. IV: 3.51, 1.80, 2.82, 3.12, 1.54, 12.79. Mid-widths (lateral): femora I–IV = 0.80, 0.85, 0.95, 0.91, palp = 0.51; patellae I–IV = 0.79, 0.84, 0.84, 0.87, palp = 0.65; tibiae I–IV = 1.04, 0.65, 0.58, 0.77, palp = 0.78; metatarsi I–IV = 0.56, 0.38, 0.26, 0.39; tarsi = 0.38, 0.37, 0.29, 0.29, palp = 0.61. Abdomen 3.96 long, 2.61 wide. Spinnerets: PMS, 0.61 long, 0.26 wide, 0.23 apart; PLS, 0.75 basal, 0.52 middle, 0.69 distal; mid-widths (lateral), 0.39, 0.34, 0.26, respectively. Carapace (Fig. 25): length to width 1.24. Fovea strongly recurved, 0.74 wide. Eyes: tubercle 0.29 high, length 0.69, width 1.07. Clypeus 0.04. Anterior and posterior eye row recurved. Eyes sizes and inter-distances: AME 0.25, ALE 0.27, PME 0.09, PLE 0.15, AME–AME 0.11, AME–ALE 0.07, PME–PME 0.50, PME–PLE 0.02, ALE–PLE 0.07, AME–PME 0.09, ALE–ALE 0.69, PLE–PLE 0.73. Eye group width 1.06, length 0.53. Maxillae (Fig. 26) 1.06 long, 1.79 wide. Cuspules: 39 spread over ventral inner heel. Labium: 0.73 long, 0.80 wide, with 3 cuspules. Labio-sternal groove shallow, flat with two large sigillae. Sternum: 2.61 long, 2.13 wide. Three pairs of sigillae, first, second rounded, posterior ovals, all one diameter from margin. Chelicerae: basal segment with 7 teeth. Legs: leg formula: I IV II III. Scopula: tarsi I–II scopulate. Metatarsus I 1/2, II 1/3 scopulate. Spines: palp: femur p0-0-1ap, patella p1, tibia 0; leg I: femur d1-1-2, p0-0-1ap, patella v2, p0-1-0, tibia v2-2-0, p0-0-1, metatarsus v0-1-1; leg II: femur d1-1-2; patella v0-0-2ap, p0-1-1ap; tibia: v2-5-2ap, p0-1-1ap, r0-0-1ap; metatarsus v2-5-2ap, p0-1-0; leg III: femur d1-2-2(1ap), patella p4, r1, tibia v2-3-2ap, d0-1-0, r1-1-0, p1-1-0, metatarsus v2-4-2ap, d3-2-2ap, r0-1-1ap, p1-2-1ap; leg IV: femur d2-1-1, patella 0, tibia v2-2-2ap, r1-0-1, metatarsus: v0-5-2ap, d0-0-1ap, r0-1-1ap, p0-1-1(1ap). Preening-comb: formed by 4–5 small spines between 2 larger spines on ventral-retrolateral tip of metatarsus IV. ITC smooth, STC with two rows of 5–6 teeth on both margins on all legs. Palp (Figs 16–17): embolus 0.77 in length. Embolus basal, middle, distal width of 0.14, 0.03, 0.01, respectively. Tegulum 0.55 long. Tibial spur (Figs 18–20) formed by single branch 0.2 long, 0.2 wide, on retrolateral margin, with apical spine. Color pattern: carapace dark brown with some long golden setae. Sternum, labium, maxillae, coxae orange brown. Abdomen black with rounded whitish spot on dorsal anterior region, brown punctuations on remaining areas. Spinnerets light brown with brown setae. All legs uniform brown.

**Figures 21–26.**
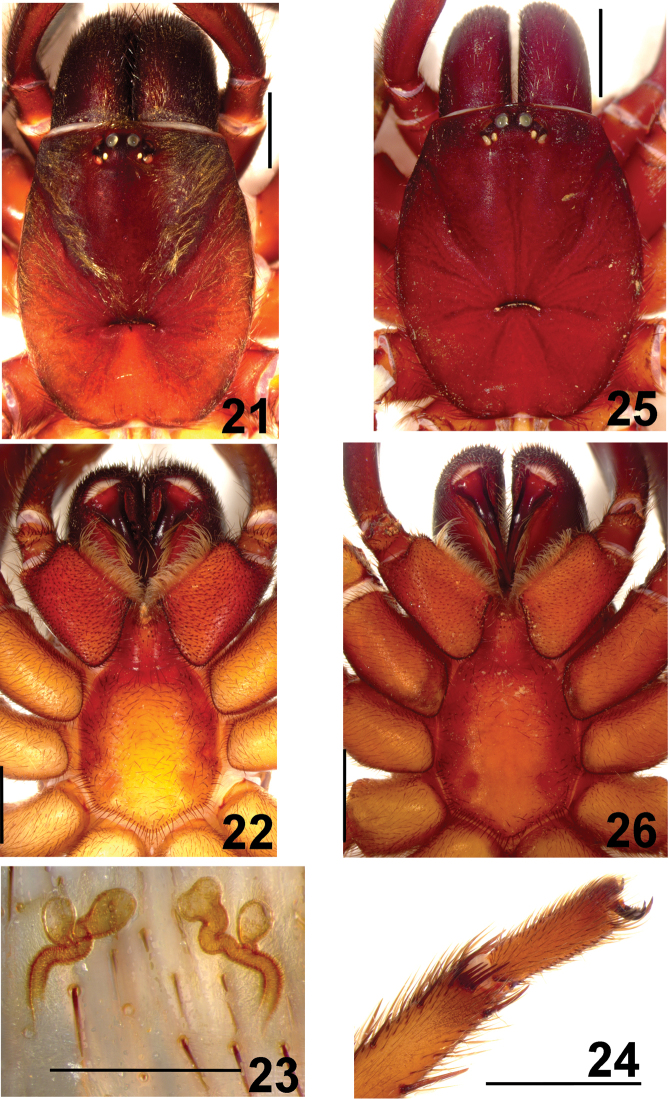
*Fufius candango* sp. n. **21–24** Paratype female **21** carapace **22** sternum, labium and maxillae **23** spermathecae, dorsal view **24** right metatarsus IV, retrolateral, showing spiniform preening-comb **25–26** holotype male **25** carapace **26** sternum, labium and maxillae. Scale bar = 1mm.

##### Female description

(DZUB 4492-1). Total length: 12.71. Carapace 5.58 long, 4.40 wide, chelicerae 1.71 long, 1.45 wide. Palp: femur 2.19, patella 1.47, tibia 1.29, tarsus 1.49, total 6.44. Legs (femur, patella, tibia, metatarsus, tarsus, total): I: 3.84, 2.35, 2.11, 2.29, 1.67, 12.26. II: 2.98, 2.16, 1.65, 2.12, 1.56, 10.47. III: 2.56, 1.83, 1.28, 1.93, 1.48, 9.08. IV: 3.88, 2.01, 2.80, 2.64, 1.30, 12.63. Mid-widths (lateral): femora I–IV = 0.94, 0.96, 1.06, 1.11, palp = 0.60; patellae I–IV = 0.90, 0.96, 0.88, 0.93, palp = 0.77; tibiae I–IV = 0.84, 0.55, 0.73, 0.75, palp = 0.61; metatarsi I–IV = 0.52, 0.48, 0.48, 0.42; tarsi = 0.52, 0.46, 0.40, 0.38, palp = 0.62. Abdomen 6.34 long, 3.56 wide. Spinnerets: PMS, 0.81 long, 0.31 wide, 0.44 apart; PLS, 1.15 basal, 0.84 middle, 0.91 distal; mid-widths (lateral), 0.62, 0.60, 0.43 respectively. Carapace (Fig. 21): length to width 1.27. Fovea recurved, 1.11 wide. Eyes: tubercle 0.41 high, length 0.80, width 1.16. Clypeus 0.16. Anterior and posterior eye row recurved. Eyes sizes and inter-distances: AME 0.30, ALE 0.26, PME 0.09, PLE 0.21, AME-AME 0.14, AME–ALE 0.10, PME–PME 0.53, PME–PLE 0.06, ALE–PLE 0.08, AME–PME 0.11, ALE–ALE 0.75, PLE–PLE 0.73. Eye group width 1.16, length 0.56. Maxillae (Fig. 22) 1.63 long, 1.91 wide. Cuspules: ca. 39 spread over ventral inner heel. Labium: 0.84 long, 1.01 wide, with 4 cuspules. Labio-sternal groove shallow, flat with two large sigillae. Sternum: 2.85 long, 2.56 wide. Three pairs of sigillae, first rounded, second, third ovals, all one diameter from margin and hardly visible. Chelicerae: basal segment with 7 teeth. Legs: leg formula: IV I II III. Scopula: tarsi I–II scopulate. Metatarsus I 1/4, II 1/3 scopulate. Spines: palp: femur p-0-0-1, patella 0, tibia v2-2-3(2ap), p0-1-0, tarsus v2-0-0; leg I: femur p0-0-1, patella p1; tibia v0-1-2ap, metatarsus v1-2-2ap; leg II: femur p-0-0-1, d1-0-1, patella p0-1-0, tibia v1-1-2ap, p0-0-1, metatarsus v1-3-2ap, p0-1-0, r0-0-1ap; leg III: femur d1-0-0; patella p7; tibia: v2-2-2ap, p1-1-1ap, r0-1-1, metatarsus d3-3-2ap, p0-0-1ap, v2-4-2(2ap), r1-1-1ap; leg IV: femur: d1-0-0, patella 0, tibia v2-2-2ap, r0-2-1(1ap), metatarsus: d0-0-1(1ap), p0-2-3(2ap), v2-2-2ap, r0-0-1ap. Preening-comb (Fig. 24): formed by 4–5 small spines between two bigger spines on ventro-retrolateral tip of metatarsus IV. Palp with single claw having 6 small teeth on internal margin. ITC smooth, STC with two rows of 8 teeth on both margins on all legs. Spermathecae (Fig. 23): two spermathecae having narrow and inward curved stalk, giving origin to two spiraled branches ending in single bulb. Color pattern: as in male, except carapace brown, cephalic area darker; ventral abdominal area with lighter portion close to spinnerets; legs black with two (femur, patella, tibia) or one (metatarsus, tarsus) brown stripes (lacking setae) on dorsal area.

##### Remarks.

The male of this species was erroneously attributed to *Fufius funebris* Vellard (1924) by Guadanucci and Indicatti (2004). See discussion for *Fufius funebris* above.

##### Distribution.

Known only from type locality: Brazil, Distrito Federal (Brasília) (Fig. 45).

#### Fufius minusculus sp. n.

http://zoobank.org/F1E97285-664F-45E8-8E2B-D793F0CBE42A

http://species-id.net/wiki/Fufius_minusculus

Figs 27 –36 , 45

##### Diagnosis.

Male and female differ from those of all other species by carapace at least 1.5 times longer than wide and small sternal sigillae (Figs 32–36).

##### Etymology.

The specific name refers to the tiny size of the species.

##### Types.

Holotype male from Brazil, *Tocantins*, Mateiros, Jalapão [10°32'S, 46°24'W], 01 November 2004, S. Balbino, with pitfall trap (DZUB 3416). Paratype: female from Brazil, *Tocantins*, Palmas, km 1.5 on the railway TO-010 (Palmas-Lajeado) [10°09'S, 48°18'W], 3 November 2001, I. Knysak & R. Martins (IBSP 9695).

##### Additional material examined.

BRAZIL: *Tocantins*: Palmas [10°10'S, 48°19'W], 2 immatures, 30 September 2001, I. Knysak & R. Martins (IBSP 9689).

##### Male description

**Figures 27–31.**
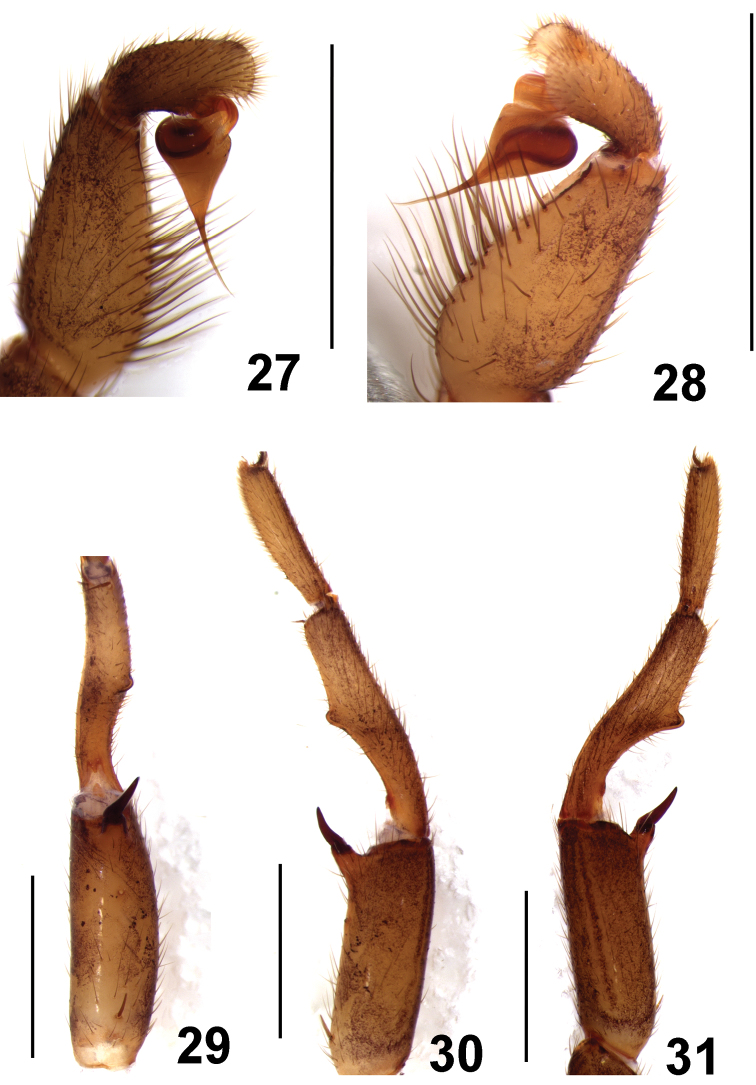
*Fufius minusculus* sp. n., holotype male **27–28** left palpal bulb **27** prolateral view **28** retrolateral view **29–31** right leg I tibial spur **29** ventral view **30** prolateral view **31** retrolateral view. Scale bar = 1mm.

(DZUB 3416). Total length: 5.11. Carapace 2.42 long, 1.60 wide, chelicerae 1.09 long, 0.60 wide. Palp: femur 1.24/ patella 0.66/ tibia 0.86/ tarsus 0.46/ total 3.22. Legs (femur, patella, tibia, metatarsus, tarsus, total): I: 1.88, 1.07, 1.39, 1.41, 0.95, 6.70. II: 1.61, 0.93, 1.31, 1.30, 0.97, 6.12. III: 1.14, 0.78, 0.70, 0.98, 0.35, 3.95. Leg IV missing. Mid-widths (lateral): femora I–III = 0.40, 0.38, 0.51, palp = 0.31; patellae I–III = 0.38, 0.32, 0.38, palp = 0.34; tibiae I–III = 0.49, 0.23, 0.30, palp = 0.44; metatarsi I–III = 0.27, 0.19, 0.18; tarsi I–III = 0.21, 0.13, 0.18, palp = 0.26. Abdomen 2.41 long, 1.29 wide. Spinnerets: PMS, 0.25 long, 0.15 wide, 0.13 apart; PLS, 0.34 basal, 0.22 middle, 0.28 distal; mid-widths (lateral), 0.26, 0.20, 0.15, respectively. Carapace (Fig. 35): length to width 1.51. Fovea slightly recurved, 0.37 wide. Eyes: tubercle 0.19 high, length 0.32, width 0.50. Clypeus 0.02. Anterior and posterior eye row recurved. Eyes sizes and inter-distances: AME 0.14, ALE 0.14, PME 0.05, PLE 0.11, AME–AME 0.05, AME–ALE 0.04, PME–PME 0.23, PME–PLE 0.02, ALE–PLE 0.03, AME–PME 0.05, ALE–ALE 0.34, PLE–PLE 0.31. Eye group width 0.54, length 0.26. Maxillae (Fig. 36) 0.59 long, 0.80 wide. Cuspules: 12 spread over ventral inner heel. Labium: 0.31 long, 0.52 wide, with 2 cuspules. Labio-sternal groove shallow, flat, sigillae not evident. Sternum: 1.44 long, 1.13 wide. Sigillae: first, second pairs small, rounded, less than one diameter from margin. Third small, oval, one diameter from margin. Chelicerae: basal segment with 6 teeth. Legs: leg formula: I II III (legs IV missing). Scopula: tarsi I–II scopulate. Metatarsi III ascopulate (leg IV missing). Spines: palp: femur p-0-0-1, patella 0, tibia 0; leg I: femur d1-0-0, patella 0, tibia v1-1-0, metatarsus v0-0-ap1, p0-0-ap1; leg II: femur d 1-0-0, patella 0, tibia v 1-1-1ap, metatarsus v1-1-1ap; leg III: femur v1-0-0, patella p4, tibia d1-0-1, r0-0-1ap, v0-1-2ap, p0-0-1ap, metatarsus d1-3-2(1ap), r0-0-1ap, v1-2-2ap, p0-1-1. Leg IV missing. ITC smooth, STC with two rows of 5–8 teeth on both margins on all legs. Palp (Figs 27–28): embolus 0.46 in length. Embolus basal, middle, distal width of 0.22, 0.02, 0.01, respectively. Tegulum 0.24 long. Tibial spur (29–31) formed by single branch 0.28 long, 0.22 wide, on retrolateral margin, with apical spine. Color pattern: carapace, chelicerae reddish brown, darker on cephalic area, carapace margin, chelicerae; sternum, labium, maxillae, coxae of legs and palp light brown; abdomen dark with light brown punctuations on dorsum, larger white spot on central anterior area. Ventrally dark with central area whitish. Spinnerets light brown with dark brown setae. Legs yellowish with black areas on distal femora, most of patellae, tibiae, metatarsi.

**Figures 32–36.**
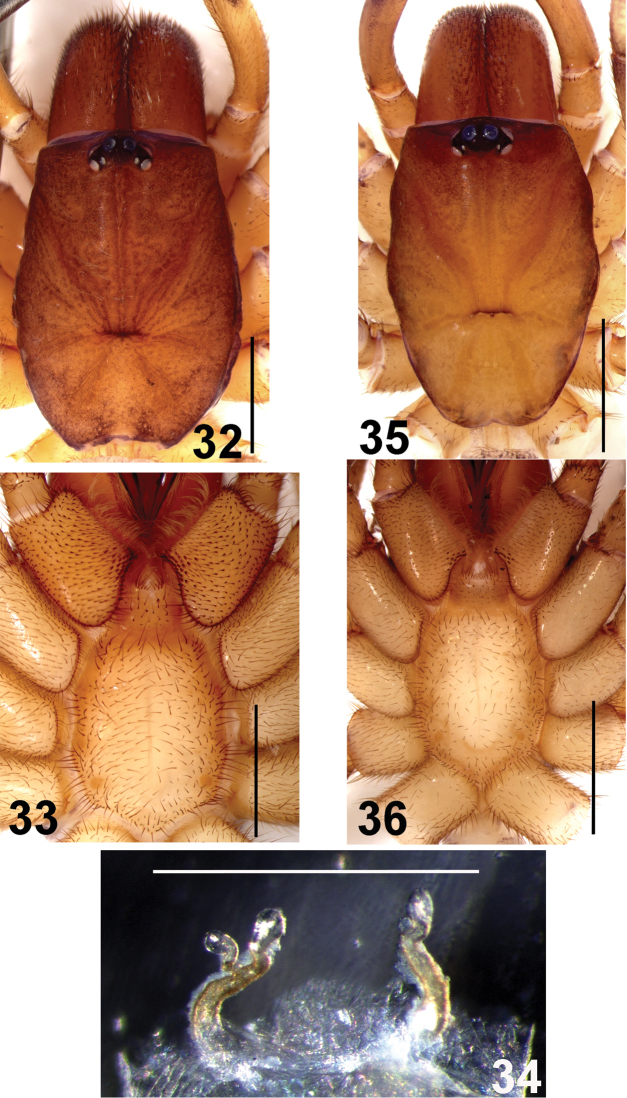
*Fufius minusculus* sp. n. **32–34** paratype female **32** carapace **33** sternum, labium and maxillae **34** spermathecae, dorsal view **35–36** holotype male **35** carapace **36** sternum, labium, and maxillae. Scale bar = 1mm.

##### Female description

(IBSP 9695). Total length: 5.19. Carapace 3.16 long, 2.18 wide, chelicerae 0.93 long, 0.75 wide. Palp: femur 1.58, patella 0.98, tibia 0.68, tarsus 0.93, total 4.17. Legs (femur, patella, tibia, metatarsus, tarsus, total): I: 1.79, 1.15, 1.20, 1.06, 0.77, 5.97. II: 1.32, 0.88, 0.85, 0.98, 0.98, 5.01. III: 1.35, 0.92, 0.64, 0.89, 0.88, 4.68. IV: 1.69, 1.09, 1.56, 1.41, 0.76, 6.51. Mid-widths (lateral): femora I–IV = 0.48, 0.51, 0.56, 0.51, palp = 0.40; patellae I–IV = 0.46, 0.46, 0.45, 0.43, palp = 0.38; tibiae I–IV = 0.44, 0.33, 0.37, 0.38, palp = 0.38; metatarsi I–IV = 0.27, 0.21, 0.23, 0.24; tarsi I–IV = 0.26, 0.22, 0.17, 0.22, palp = 0.29. Abdomen 3.36 long, 2.40 wide. Spinnerets: PMS, 0.40 long, 0.23 wide, 0.35 apart; PLS, 0.65 basal, 0.51 middle, 0.49 distal; mid-widths (lateral), 0.34, 0.28, 0.26, respectively. Carapace (Fig. 32): length to width 1.45. Fovea slightly recurved, 0.45 wide. Eyes: tubercle 0.15 high, length 0.46, width 0.62. Clypeus 0.07. Anterior and posterior eye row recurved. Eyes sizes and inter-distances: AME 0.17, ALE 0.18, PME 0.08, PLE 0.11, AME 0.05, AME–ALE 0.04, PME–PME 0.27, PME–PLE 0.03, ALE–PLE 0.03, AME–PME 0.07, ALE–ALE 0.45, PLE–PLE 0.40. Eye group width 0.63, length 0.29. Maxillae (Fig. 33) 0.72 long, 0.93 wide. Cuspules: ca. 30 spread over ventral inner heel. Labium: 0.45 long, 0.55 wide, with 2 cuspules. Labio-sternal groove shallow, flat, sigillae not evident. Sternum: 1.60 long, 1.23 wide. Sigillae: first, second pairs small, rounded, less than one diameter from margin. Third small, oval, one diameter from margin. Chelicerae: basal segment with 7 teeth. Legs: leg formula: IV I II III. Scopula: tarsi I–II scopulate. Metatarsi I 1/3 scopulate. Spines: palp: femur 0, patellae r1-1-0, tibia v2-2-3ap, r1-1-1ap, p0-0-1, tarsus v2-0-0; leg I: femur p0-0-1ap, patellae p0-0-1ap, tibia v1-1-1ap, metatarsus v1-1-2ap; leg II: femur 0, patellae r1-1-0, tibia v1-1-2, metatarsus v0-3-3(2ap), p0-1-0, r0-0-1; leg III: femur 0, patellae p4, r1, tibia d1-0-0, r0-1-0, v0-2-2ap, p1-1-0, metatarsus d1-3-2ap, r0-0-1, v0-4-2ap, p0-1-0; leg IV: femur 0, patella 0, tibia v0-1-2, r0-1-1, metatarsus d0-0-1ap, r0-1-0, v0-2-1ap, p1-1-1ap. Preening-comb: absent on retrolateral tip of metatarsus IV. Palp with single claw having 8 small teeth on internal margin. ITC smooth, STC with two rows of 5–9 teeth on both margins on all legs. Spermathecae (Fig. 34): two spermathecae having narrow and inward curved stalk, giving origin to two sinuous branches ending in single bulb each. Color pattern: as in male.

##### Distribution.

Brazil, state of Tocantins (Palmas and Mateiros) (Fig. 45).

#### Fufius jalapensis sp. n.

http://zoobank.org/8D27137C-9415-45B0-A01A-5B79248E1C4C

http://species-id.net/wiki/Fufius_jalapensis

Figs 37 –43 , 45

##### Diagnosis.

Male differs from those of all other species by tibia I lacking spines (Figs 39–41) and the presence of several spines (ca. 11) on the prolateral patella III. The female is unknown.

##### Etymology.

The specific name, jalapensis, refers to the type locality, “Jalapão”, a state park in the eastern state of Tocantins, Brazil.

##### Types.

Holotype male from BRAZIL, *Tocantins*: Mateiros, Jalapão [10°32'S, 46°24'W], 01 November 2004, S. Balbino (DZUB 3370). Paratype male, same collector and date (DZUB 4469).

##### Male description

(DZUB 3370). Total length: 11.72. Carapace 5.92 long, 4.61 wide, chelicerae 1.89 long, 1.39 wide. Palp: femur 2.88, patella 1.46, tibia 2.09, tarsus 0.83, total 7.26. Legs (femur, patella, tibia, metatarsus, tarsus, total): I: 4.47, 3.15, 3.86, 3.71, 2.35, 17.54. II: 3.44, 2.63, 3.33, 3.29, 2.03, 14.72. III: 2.95, 1.91, 2.14, 3.08, 1.84, 11.92. IV: 4.33, 2.47, 4.55, 4.24, 1.83, 17.42. Mid-widths (lateral): femora I–IV = 0.89, 0.80, 1.14, 1.21, palp = 0.47; patellae I–IV = 0.80, 0.70, 0.86, 0.87, palp = 0.78; tibiae I–IV = 0.89, 0.57, 0.64, 0.79, palp = 0.87; metatarsi I–IV = 0.61, 0.44, 0.38, 0.45; tarsi = 0.49, 0.45, 0.40, 0.33, palp = 0.55. Abdomen 5.51 long, 3.27 wide. Spinnerets: PMS, 0.60 long, 0.28 wide, 0.15 apart; PLS, 0.88 basal, 0.49 middle, 0.81 distal; mid-widths (lateral), 0.52, 0.44, 0.33 respectively. Carapace (Fig. 42): length to width 1.28. Fovea strongly recurved, 1.29 wide. Eyes: tubercle 0.24 high, length 0.64, width 1.14. Clypeus 0.07. Anterior and posterior eye row recurved. Eyes sizes and inter-distances: AME 0.30, ALE 0.28, PME 0.10, PLE 0.22, AME–AME 0.06, AME–ALE 0.10, PME–PME 0.60, PME–PLE 0.04, ALE–PLE 0.14, AME–PME 0.13, ALE–ALE 0.73, PLE–PLE 0.80. Eye group width 1.11, length 0.54. Maxillae (Fig. 43) 2.06 long, 1.32 wide. Cuspules: 31 spread over ventral inner heel. Labium: 0.73 long, 1.0 wide, with 3 cuspules. Labio-sternal groove shallow, flat with two large sigillae. Sternum: 3.04 long, 2.54 wide. Sigillae: first pair rounded, second, third ovals, all less than one diameter from margin. Third pair large, twice the diameter of second. Chelicerae: basal segment with 7 teeth. Legs: leg formula: I IV II III. Scopula: tarsi I–III scopulate. Metatarsi I 1/5, II 1/3 scopulate. Spines: palp 0; Leg I 0; Leg II: femur, patella 0, tibia v1-1-2ap, p0-0-1ap, metatarsus v0-2-1ap, r0-0-1ap; Leg III: femur 0, patella p11, tibia d0-1-0, v0-1-1ap, p0-1-2(1ap), metatarsus d1-3-1ap, r0-0-1ap, v2-3-2ap, p2-1-3(2ap); Leg IV: femur, patellae 0, tibia r0-0-1, v0-1-0, metatarsus r0-1-1ap, v1-3-1ap, p1-0-2ap. Preening-comb: formed by 6 spiniform setae between two spines on ventro-retrolateral tip of metatarsus IV. ITC smooth, STC with two rows of 3–9 teeth on both margins on all legs. Palp: embolus 0.82 in length. Embolus basal, middle, distal width of 0.28, 0.05, 0.02, respectively. Tegulum 0.36 long. Tibial spur formed by single branch 0.54 long, 0.36 wide, on retrolateral margin, with apical spine. Color pattern: carapace, chelicerae brown, darker on cephalic area, carapace margin, chelicerae; sternum, labium, maxillae, coxae of legs and palp light brown; abdomen dark with light brown punctuations on dorsum, a larger white spot on central anterior area, ventrally dark with book-lung area whitish. Spinnerets light brown with dark brown setae. Legs an almost homogeneous brown.

**Figures 37–41.**
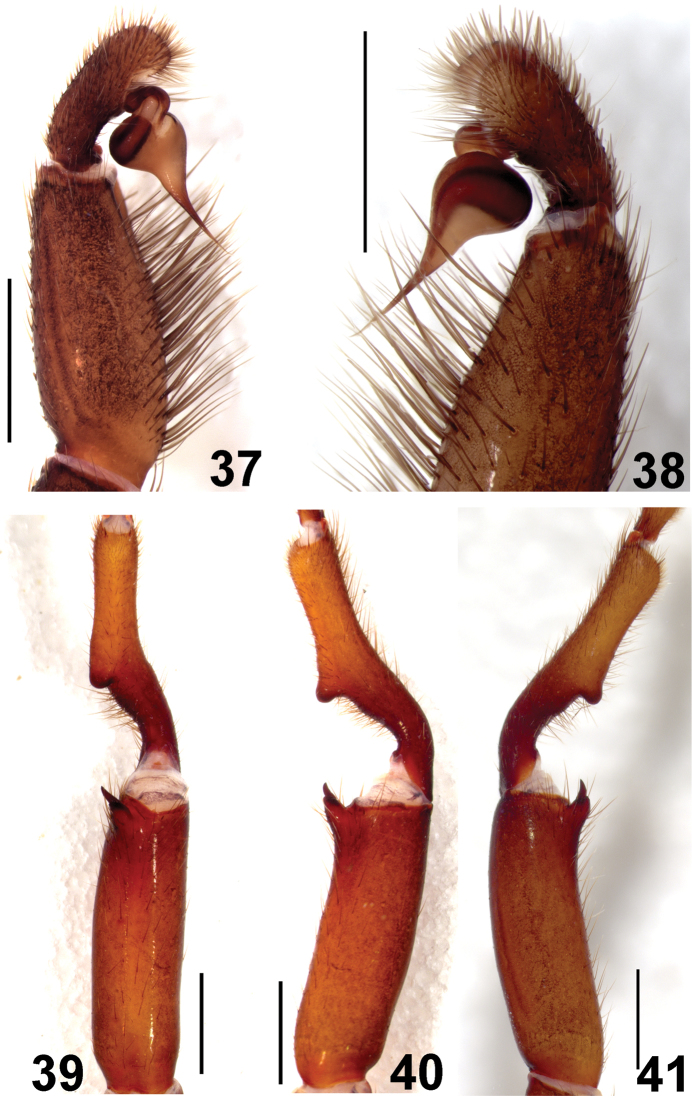
*Fufius jalapensis* sp. n., holotype male **37–38** left palpal bulb (mirrored) **37** retrolateral view **38** prolateral view **39–41** right leg I tibial spur **39** ventral view **40** prolateral view **41** retrolateral view. Scale bar = 1mm.

**Figures 42–43.**
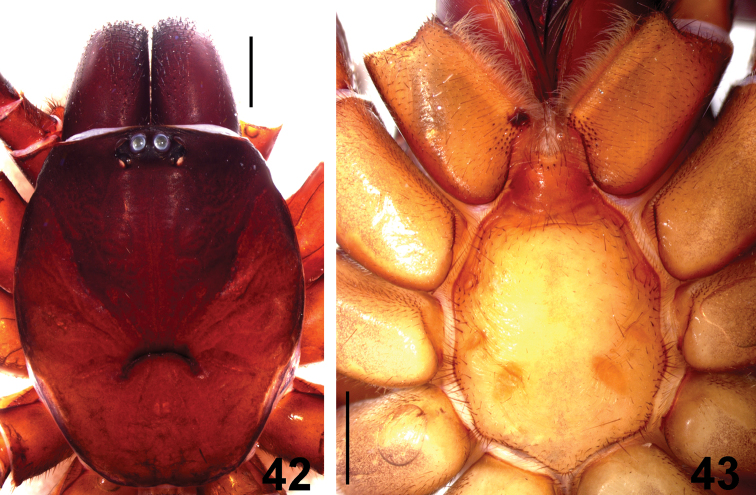
*Fufius jalapensis* sp. n., holotype male. **42** carapace **43** sternum, labium, and maxillae. Scale bar = 1 mm.

##### Female.

Unknown.

##### Distribution.

Known only from the type locality, Brazil, state of Tocantins (Jalapão region) (Fig. 45).

**Figure 44.**
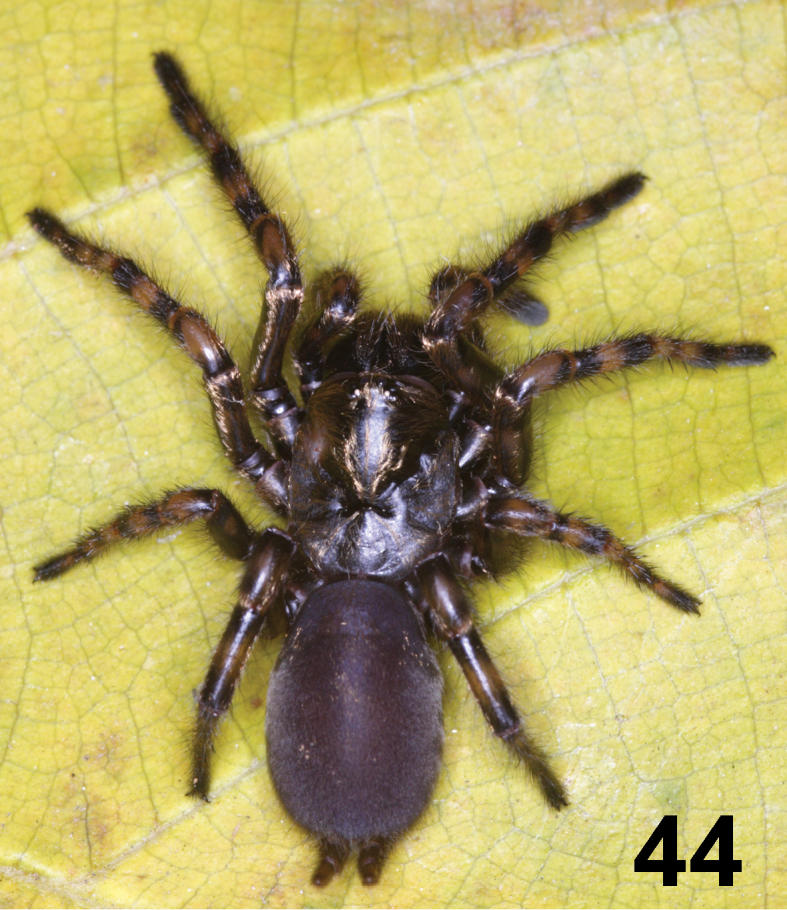
*Fufius lucasae*, female from Itapecerica da Serra, state of São Paulo. Photo: R. Bertani.

**Figure 45.**
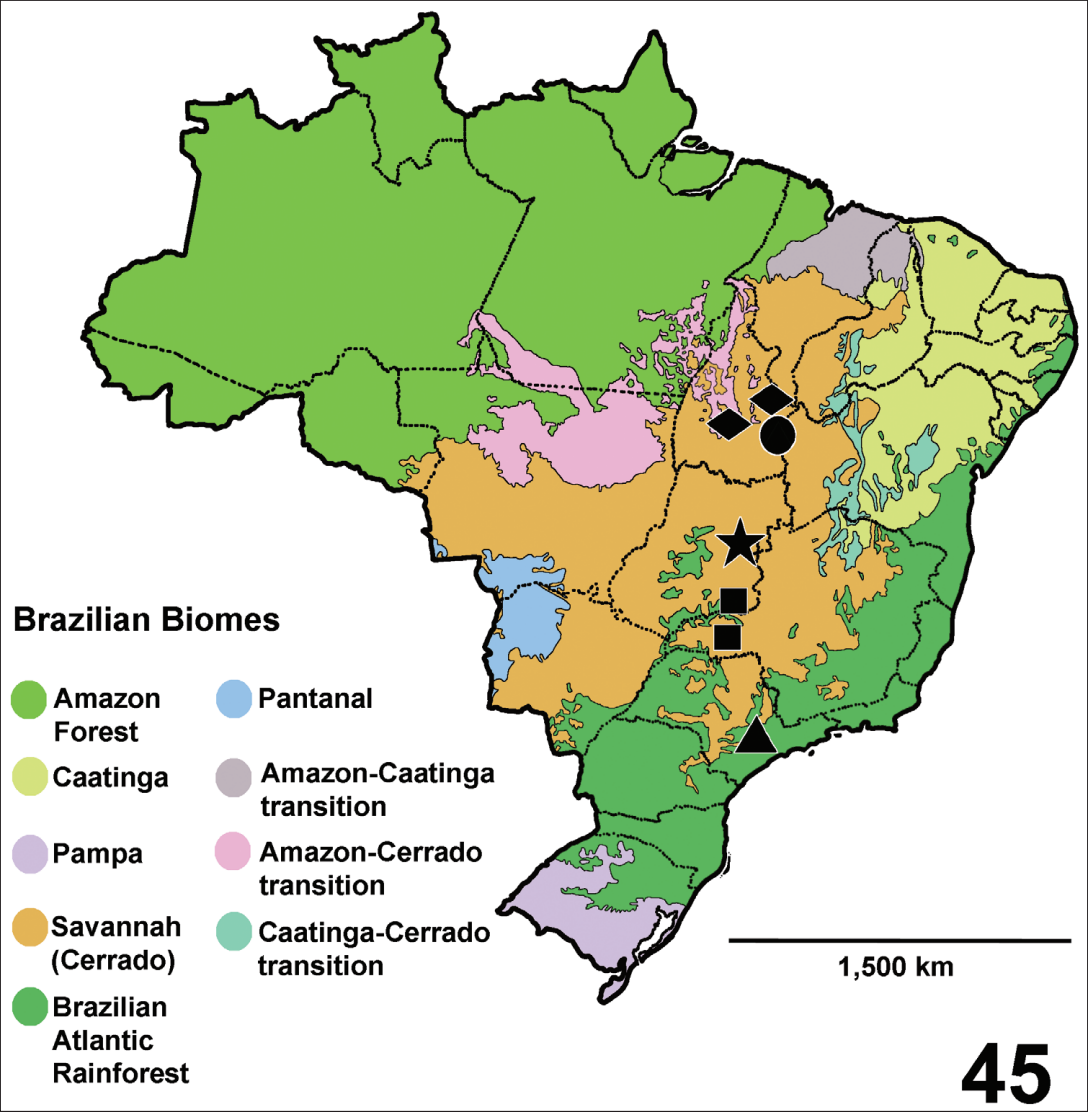
Map showing records of *Fufius funebris* (square), *Fufius lucasae* (triangle), *Fufius candango* sp. n. (star), *Fufius minusculus* sp. n. (diamond) and *Fufius jalapensis* sp. n. (circle) in different Brazilian Biomes.

## Supplementary Material

XML Treatment for Fufius funebris

XML Treatment for Fufius lucasae

XML Treatment for Fufius candango

XML Treatment for Fufius minusculus

XML Treatment for Fufius jalapensis
